# Editorial: Biointeractions among host plant, wood borers and pathogens/their associated microbes

**DOI:** 10.3389/fpls.2024.1347776

**Published:** 2024-02-01

**Authors:** Lilin Zhao, Jianghua Sun, Kathryn Bushley

**Affiliations:** ^1^State Key Laboratory of Integrated Management of Pest Insects and Rodents, Institute of Zoology, Chinese Academy of Sciences, Beijing, China; ^2^CAS Center for Excellence in Biotic Interactions, University of Chinese Academy of Sciences, Beijing, China; ^3^College of Life Science, Institute of Life Science and Green Development, Hebei University, Hebei, China; ^4^Department of Plant and Microbial Biology, University of Minnesota, Saint Paul, MN, United States

**Keywords:** wood borer, bark beetle, biointeraction, pathogenic fungi, woody plant

Global forests face increasingly difficult challenges and many are attributed to the presence of wood boring beetles and pathogens. Fueled by globalization, the outbreak of forest pests and diseases results from a complex interplay among wood borers, pathogens, and plants. Within these intricate interrelationships, key nodes such as the distinct physiological characteristics of pests at different life stages, synergistic interactions between pests and pathogenic fungi, host organisms’ immune responses to pathogenic fungi, and resistance of hosts can be identified as novel targets for ecological control measures. To further contribute to the theoretical framework for the prevention and control of forestry pests and diseases, this Research Topic of biointeractions among host plants, wood borers, and pathogens or their associated microbes has gathered six original research articles focusing on these key nodes.

Spruce trees have faced severe outbreaks of bark beetles belonging to the *Ips* genus (Coleoptera: Curculionidae, Scolytinae) (Mandelshtam and Selikhovkin, 2020). The bark beetle *I. typographus* undergoes a complex life cycle with two distinct adult forms: callow and sclerotized. Callow beetles consume food voraciously, while sclerotized males display increased aggression and lead the initiation of new colonies. Ashraf et al. revealed that gut enzymes, including NADPH-cytochrome P450 reductase and glutathione S-transferase, are pivotal for detoxification and digestion of host-plant allelochemicals. Upregulation of chitin metabolism and juvenile hormone biosynthesis-associated proteins in callow beetles and the tricarboxylic acid cycle and fatty acid metabolism-associated proteins in sclerotized beetles showed the adaptability of *I. typographus* at different stages. This study will facilitate future research at the protein level on beetle pests, offering insights for developing targeted forest pest management strategies through the targeting of essential proteins.

During the process of bark beetle infestation on spruce trees, the associated pathogenic fungi form a complex with the bark beetle, aiding it in overcoming tree defenses and creating a suitable environment for beetle offspring (Franceschi et al., 2005). Through infestation on *Picea jezoensis* and *Larix olgensis* with two bark beetles (*I. typographus* and *I. subelongatus*) and their associated pathogenic fungi (*Endoconidiophora polonica* and *E. fujiensis*), Shi et al. disclosed a species-specific association existing between the beetles and fungi. The bark beetle-associated pathogenic fungus complex *I. typographus*-*E. polonica* demonstrated exceptional host compatibility with *P. jezoensis* but failed to colonize or infect *L. olgensis*. The complex *I. subelongatus*-*E. fujiensis* exhibited robust host compatibility with *L. olgensis* and a certain level of compatibility with *P. jezoensis*. This study confirms that the beetle-fungus complex *I. typographus*-*E. polonica* and *I. subelongatus*-*E. fujiensis* both demonstrated optimal performances on their respective traditional hosts, with the *I. subelongatus*-*E. fujiensis* complex displayed a broader range of host suitability. This study provides, for the first time, biochemically derived information on the consistent suitability of bark beetles and their associated pathogenic fungi for host plants.

During the long-term co-evolution of the bark beetles and fungi complex, beetles have been found capable of carrying Ophiostomatoid fungi. Traditionally, these fungi were thought to deplete the defenses of the host tree (Six, 2012). Liu et al. uncovered that *Ophiostoma bicolor* could induce monoterpenoid release from spruce and upregulate the expression of genes involved in both monoterpene precursor and diterpene synthesis. This suggests that *O. bicolor* potentially induces host defense rather than depleting it. The finding challenges the traditional notion of defense depletion and suggests a nuanced interplay between the host and fungal invaders, paving the way for a deeper understanding of spruce defense mechanisms.

In addition to the bark beetle and fungus complex, there are many other wood borer species that either transport assemblies of phytopathogens or maintain close associations with specific partners, exacerbating the adverse impacts of their insect hosts and, in some cases, leading to substantial forest collapses (Linnakoski and Forbes, 2019). Through investigating entomopathogenic fungi in pine sawyer beetle *Monochamus alternatus* from various geographical populations, Wu et al. identified 640 fungal strains. Notably, certain fungi exhibited strong entomopathogenic activities, while others displayed phytopathogenic potential. These findings suggest a potential biocontrol factor of pine sawyers in pine forests. This study significantly enhances the exploration of valuable control resources for pine sawyer beetles.

Faced with the biotic stresses from pests and pathogens, plants have evolved complex defense systems. Salicylic acid (SA) in poplar trees has been reported to be closely associated with disease resistance (Ullah et al., 2019; Xiao et al., 2021). It can undergo conversion into methyl salicylate (MeSA) by the enzyme salicylic acid methyltransferase (SAMT) and, conversely, can be transformed back into SA by SA-binding protein 2 (SABP2). In the investigation of poplar response to the pathogenic fungus *Botryosphaeria dothidea*, Dong et al. observed a substantial increase in MeSA content, a noticeable decrease in SA content, and heightened SAMT expression, while poplar showed upregulation of pathogenesis-related genes PR-1 and PR-5, susceptibility to *B. dothidea* increased. This research contributes to a better understanding of the coordinated regulation between SAMT and SABP2 in poplar, emphasizing the maintenance of a balance between SA and MeSA to enhance defense against pathogen invasion.

Disease resistance is essential for the health of plants. Gummy stem blight (GSB) emerges as a formidable threat to cucurbits production, and several GSB-resistant Quantitative Trait Loci (QTLs) have been reported (Lee et al., 2021). However, the elusive causal genes remained unidentified in cucumber. For fine genetic mapping, Han et al. developed a strategy by utilizing residual heterozygous lines and the location of a novel locus, gsb3.1, responsible for seedling GSB resistance, was narrowed down to a 38 kb interval. Candidate genes within this locus show promise for advancing genetic studies and marker-assisted selection, potentially offering broad-spectrum GSB resistance in cucumber. This study will provide insights into the genetic mechanisms underlying plant resistance, which can inform potential mechanisms of forest tree resistance.

Studies in this topic have provides valuable strain resource information for the control of pine sawyer beetles, offered a new strategy for screening resistant plants, and made theoretical progress in understanding the pest bark beetle and associated pathogenic fungi of spruce, as well as the resistance mechanism of poplar and cucumber. The theoretical advancements include: 1) The gut protein expression of bark beetles is correlated with varying adaptability at different stages; 2) The bark beetle maintains a stable species-specific association with the associated pathogenic fungi; 3) Ophiostomatoid fungi induced the host spruce’s defense mechanisms rather than depleting it; and 4) The regulation of SA content is more important than PR genes in poplar resistance. These efforts collectively contribute to advancing our comprehension regarding the interactions among pests, pathogens, and host plants, offering potential biocontrol strategies for the management of forest pests and diseases and enhancing forest resilience against devastating pests and diseases.

## Author contributions

LZ: Writing – original draft, Writing – review & editing. JS: Writing – review & editing. KB: Writing – review & editing.
